# The Threatened Strike of Asylum Nurses

**Published:** 1913-10-25

**Authors:** 


*To the Editor of* The Hospital.

### "The Threatened Strike of Asylum Nurses."

*To the Editor of* The Hospital.

Sir,—With astonishment I have read the article on page 54 of your issue for October 11 dealing with "The Threatened Strike of Asylum Nurses." The first column of your article is in the main correct, but the second column is not only an insult to the large body of women employed as mental nurses, but it is largely inaccurate, and has surely been written by someone ignorant of the true state of affairs I speak from experience as an old mental nurse and mother of one now engaged in mental work. I look back and think how many of my fellow-workers in days gone by succeeded in life; several became matrons in large institutions; others married well and filled high positions with grace and dignity.

In all large hospitals—and I have worked in some of the largest—you will find here and there an underbred woman. I am speaking now of general as well as mental work; but why on that account proceed to wholesale condemnation? The mental nurses with whom I worked were in every respect the equals by birth and education of those I had as colleagues in general work. Indeed, it is amusing to find The Hospital on page 26 of this same issue inviting asylum workers to contribute articles on their work. I agree with you as to the folly of a strike of asylum nurses; indeed, I cannot conceive such an idea being seriously entertained, and I feel certain that the majority of those engaged in mental work would shrink from the suggestion of such a movement. But when you say such a movement would be impossible in a hospital or group of hospitals I would fain point out that one reason for this is the overcrowded state of the nursing profession, so far, at any rate, as general work is concerned. Nursing is the stand-by of the poor gentlewoman. So many of them are too imperfectly educated to earn a living by teaching, they shrink from the publicity of shop work, and nursing is, they consider, "genteel." Therefore, they enter into competition with others already on the list for appointments in general hospitals. Only in mental work or in hospitals dealing with infectious diseases is the demand larger than the supply. If the nursing staff of any given large general hospital went on strike to-morrow their places would be quickly and easily filled; but with infectious diseases or mental work matters are different. The "genteel" person shrinks from mental nursing partly from fear, largely from ignorance; but did the nursing profession generally know more of mental work, more of its bright side and the independence it offers compared with general work, they would, I am confident, take it as a branch of their training.

Over thirty years ago I left a district asylum to take up work in a large hospital. I left a place where I had been very happy under a wise and good matron, where I had a comfortably furnished bedroom for my own use. I found I had to share a bedroom with another nurse and had to deal with a matron who was a vulgar, petty tyrant. From there I went to a large fever hospital, where I had to share a room with other three nurses. I certainly came to the conclusion that mental work was far in advance of any other branch of nursing.—Yours faithfully,

October 15, 1913. M. W. J.

[We have taken the liberty of suppressing certain vigorous passages in this letter, which the writer would, we feel sure, have felt upon reconsideration were too strongly expressed.—Ed. The Hospital.]

